# Correction: Pruvost, M.; Moyon, S. Oligodendroglial Epigenetics, from Lineage Specification to Activity-Dependent Myelination. *Life* 2021, *11*, 62

**DOI:** 10.3390/life14050620

**Published:** 2024-05-11

**Authors:** Mathilde Pruvost, Sarah Moyon

**Affiliations:** Neuroscience Initiative Advanced Science Research Center, CUNY, 85 St Nicholas Terrace, New York, NY 10031, USA; mpruvost@gc.cuny.edu

## Error in References

The authors wish to make the following corrections to this paper [1]. Due to editorial formatting changes following proofreading, the numbering of references (after reference [84] in the original publication) and the number of listed references (two references missing in the original publication) have been modified. The mistakes were caused during copy editing and affected the order of references.

In the original publication [1], the reference “Nagao, M.; Lanjakornsiripan, D.; Itoh, Y.; Kishi, Y.; Ogata, T.; Gotoh, Y. High Mobility Group Nucleosome-Binding Family Proteins Promote Astrocyte Differentiation of Neural Precursor Cells. *Stem Cells* **2014**, *32*, 2983–2997. https://doi.org/10.1002/stem.1787.” was deleted by mistake from the references list during the final editorial preparation and disrupted the references number. The citation has now been inserted in the references list at the end of the review, and accurately relates to the corresponding text in the first paragraph of Section 3.3. Chromatin Reorganization during the Differentiation from NSCs to OPCs as Ref. [109]. The reference “Wang, C.-Y.; Deneen, B.; Tzeng, S.-F. MicroRNA-212 Inhibits Oligodendrocytes during Maturation by down-Regulation of Differentiation-Associated Gene Expression. *J. Neurochem.* **2017**, *143*, 112–125. https://doi.org/10.1111/jnc.14138.” was also deleted by mistake from the references list during the final editorial preparation. This citation has now been inserted in the references lists and accurately relates to the corresponding text in Table 3 as Ref. [166].

With this correction, the order of some references has been adjusted accordingly. 

## Additional Affiliation and Correspondence Information Update

In the published publication [1], there was an error regarding the affiliation for Sarah Moyon. In addition to affiliation original, the updated present affiliation should include: Current address: Institute of Neurophysiopathology, Aix Marseille University, 27 bd Jean Moulin, 13385 Marseille, France. Correspondence information updated into: Correspondence: sarah.moyon@univ-amu.fr; Tel.: +33-4-9132-4733.

The authors state that the scientific conclusions are unaffected. This correction was approved by the Academic Editor. The original publication has also been updated.
